# Applying novel economic simple green sample preparation procedures on natural and industrial specimens for chromatographic determination of insecticidal residues

**DOI:** 10.1038/s41598-023-33421-7

**Published:** 2023-05-03

**Authors:** Amira M. Hegazy, Hamada M. Mahmoud, Mohamed A. Elsayed, Nouruddin W. Ali, Rehab M. Abdelfatah

**Affiliations:** 1 Pharmaceutical Analytical Chemistry Department, Faculty of Pharmacy, Beni-Suef University (PIC #: 933339821), Beni-Suef, Egypt; 2 Zoology Department, Faculty of Science, Beni-Suef University, Beni-Suef, Egypt; 3 Pharmaceutical Analytical Chemistry Department, Faculty of Pharmacy, Fayoum University, Fayoum, Egypt

**Keywords:** Drug discovery, Plant sciences, Natural hazards, Health care, Chemistry

## Abstract

Spraying a tertiary blend of the insecticides (hexythiazox, imidacloprid, and thiamethoxam), on tomato fruits, is a routine in agriculture-attentive countries. A simple green sample preparation technique was developed and applied to the field samples. Specific HP-TLC and RP-HPLC methodologies are established to estimate the residual insecticides and applied to the prepared field specimens. In the planner chromatographic methodology, methanol:chloroform:glacial acetic acid:triethyl amine (8.5:1.5:0.2:0.1, v/v) is recommended as a mobile system. The other one is columnar chromatography; acetonitrile: water (20:80, v/v), pH 2.8, is recommended as a mobile system. The validation parameters were examined following the ICH rules. The means percentages and standard deviations of the accuracy of the HP-TLC method for the determined compounds were 99.66 ± 0.974, 99.41 ± 0.950, and 99.89 ± 0.983, correspondingly. The values were 99.24 ± 0.921, 99.69 ± 0.681, and 99.20 ± 0.692, correspondingly, when they were determined by the RP-HPLC method. The relative standard deviation percentages of the methods’ repeatability and intermediate precision ranged from 0.389 to 0.920. Both methods were highly specific having resolution factors of ≥ 1.78 and selectivity factors of ≥ 1.71. They were applied to the field samples perfectly.

## Introduction

Pharmaceutical care does not matter only the medicines, but it considers any chemical or natural substance that may affect human health too. One of these substance categories is insecticides, especially those related to direct contact with our natural nutrition. The most form of close, common, and frequent exposure to insecticides is the ingestion of food contaminated by these dangerous chemicals. Fresh or processed tomatoes are the most common constituent in our dining tables. Not only is the estimation of residual insecticides critical in labs of official institutions in the health sector but also inlabs on exporting-importing boundaries between countries. That may reflect the hygienic and economic impact of establishing selective methods of monitoring for a specific blend of insecticides on certain field samples.

The development of specific methods of analysis for a mixture of little definite insecticides and/or fungicides is a new trend in residual assessment for food and environmental analysis^1–3^. The authors have been working consistently since early 2017 using this strategy starting with extensive studies concerning specific insecticide residues on cucumbers^4,5^. And here, we are following the same concern extending our work on a different blend of the insecticides^6,7^ and the concerned substances [hiexythiazox (HTX), imidacloprid (IDD), and thiamethoxam (TTM)] by spectrophotometric techniques^8^. In this work, the authors, superiorly, present more specific techniques using separating conditions and tools. The chromatographic analysis was suggested for the assessment of the considered blend constitutions which are used uniquely in a mixture form for the protection of tomatoes, in the Middle East area, the host of this research^9^. Worldwide, the official inspecting institutions contribute great responsiveness to the estimation of residual insecticides on field samples. In this work, the authors offer separation techniques for the detection of the residual insecticides (HXT, IDD & TTM) on tomato samples with simple and short procedures of sampling (sample preparation and extraction).

Chemical structures and molecular weights of the proposed compounds; hexythiazox, Imidacloprid, and thiamethoxam are illustrated in the PubChem^10–12^. The organic configuration of the substance's molecules is figured out in Figure S1. Some methods have been found to determine them individually^13–16^. No single method has been found for analysis of the studied blend in publication except a GC method, which makes a general estimation of a huge number of insecticide residues in different parts of the tomato plant^17^. But the proposed methods, superiorly, are more specific since they determine only the specific blend of insecticides used for tomatoes. Also, we followed more economical sample preparation as simple tools and less complicated extraction procedures were used. The proposed RP-HPLC is more accessible in application than the published GC method due to its wide availability in general and official laboratories. As well, the advantage of HP-TLC over the published method is its simplicity and cost cut-off. The proposed methods are simpler, faster, and more economical than the published works. That makes them better selections and significantly valuable in quality control laboratories of developing countries.

The novelty of the work is hidden in the way the analyst prepared the pre-analysis samples "peeling off the spray-exposed surface is only the samples without interfering of the whole matrix". This way is faster and greener than the traditional QuEChERS method. Supporting the greenness and financial cut concepts of this work, the proposed sample preparation steps consume much lesser volumes of organic solvents than those used in liquid–liquid extraction and they cost lesser than the solid phase extraction. All that makes the proposed sampling and assays more recommended for local governmental inspection and guarantee.

The two main international organizations that control and regulate insecticide residue analysis^18,19^, describe methods of the data validation intended for proving obedience to upper residual allowance or control of the customer contact with insecticides. One of the main criteria is the matrix effect. Upon studying the European Commission validation set and comparing it to the corresponding items in ICH validation rules, we have found many similarities. Considering the European Commission statements in [C25] and [C26], Hegazy et al. used the standard addition technique in methods of validation procedures and collected field samples according to SANTE document guidelines^19^.

## Methodology

### Apparatus

CAMAG TLC scanner, High-performed TLC Alum sheets plated with 60 F254 (0.25 mm) (Merck, DE), and R201 Shang. Shen. Biot. Lim. Co. Camag-Linomat IV applicator were instrumentations in HP-TLC methodology.

1200 infinity series LC (Agilent Technologies), 1260 infinity UV–VIS detector (Agilent Technologies), and Eclipse plus C8 column (15, 4.6 and 5 µm) were instrumentations in RP-HPLC methodology.

### Authentic powders

HTX, IDD, and TTM (99.10%, 99.10% and 99.30%) were supplied via Sigma, EG.

### Trade powders

Macomite^®^ (HTX; 1:10, Bch. # 1019), Imdamex^®^ (IDD; 7: 10, Bch. # 3018721), and Pelxam^®^ (TTM; 2.5: 10, Bch. # 1625) were supplied via producers; Nipon Sod Co., Ltd (JP), Agrsmart Ind. (Behera, EG) and Barat Insec., Ltd (IN), respectively.

### Solvents

Methanol, acetonitrile, and acetone of HPLC grade were supplied from Sigma Chemie (GmbH, DE). Chloroform, glacial acetic acid, orthophosphoric acid, sod. sulfate and triethyl amine (analytical grade) were obtained from El-Nasr Pharm. Chem. Co. (EG).

### Sampling

#### Samples of standard solutions

Standard solutions of HTX, IDD, and TTM in concentrations of (103 µg/mL) were prepared, separately, and diluted to final concentrations of (102 µg/mL) in methanol.

Macomite^®^, Imdamex^®^, and Pelxam^®^ stock solutions (103 µg/mL) and working solutions (102 µg/mL) were prepared in methanol.

#### Samples for method validation

Successions of sequential concentration of HTX, IDD, and TTM solutions, in concentrations of 0.05–0.31, 0.20–2.0, and 0.1–1.0 µg/mL, correspondingly, were prepared.

#### Field samples

A bushel of tomatoes (≈ 10 kg) was picked up from the investigator's private land at El-Fyoum governorate, EG, during the cold weather period (≈ 20 °C). It was divided into four sets of 3 kg/set. The insecticides blend was applied on only three sets, complying with the SANTE document rules^19^, and the left set was treated as a control. Each set was re-divided into three replicates each of 1 kg.

### Preparation of the field samples

Simply, the fruits of tomatoes were peeled carefully via a pelamatic fruit peeler then the peels were chopped, thoroughly. In a centrifuge tube, the sample of peel pieces was shaken with acetonitrile (20 mL), thoroughly, and re-shaken after the addition of sod. sulfate (5 g), then centrifuged. The quantitatively collected extract was concentrated to a few milliliters under vacuum at room temperature, then diluted with acetonitrile to get 5 mL, accurately.

### Method validation

Sequential solutions of HTX (0.05–0.31 µg/mL), IDD (0.20–2.0 µg/mL), and TTM (0.1–1.0 µg/mL) were applied for achievement of HP-TLC and RP-HPLC methods linearity. The mixture solutions of them were applied to prove the precision of the methods. Limits of detection and quantitation were estimated. Both methods were applied to the trade solutions and field samples for confirmation of their applicability.

## Results and discussion

### HP-TLC methodology optimization

Many mobile systems were eluted to succeed in the complete separation resolution for the ternary mixture. Elusion of methanol:chloroform (2:8, v/v) and hexane:methanol (8:2, v/v) binary solvent mixture gave lousy separation. A tertiary solvent mixture of methanol:chloroform:acetic acid (8:2:0.2 v/v) gave better break and tailed peaks. The replacement of acetic acid with triethyl amine in the last mixture gave perfect peaks' break. The mobile system, methanol:glacial acetic acid:chloroform:triethyl amine (8.5:0.2:1.5:0.1) is selected as the method's mobile phase.

**Figure 1:**
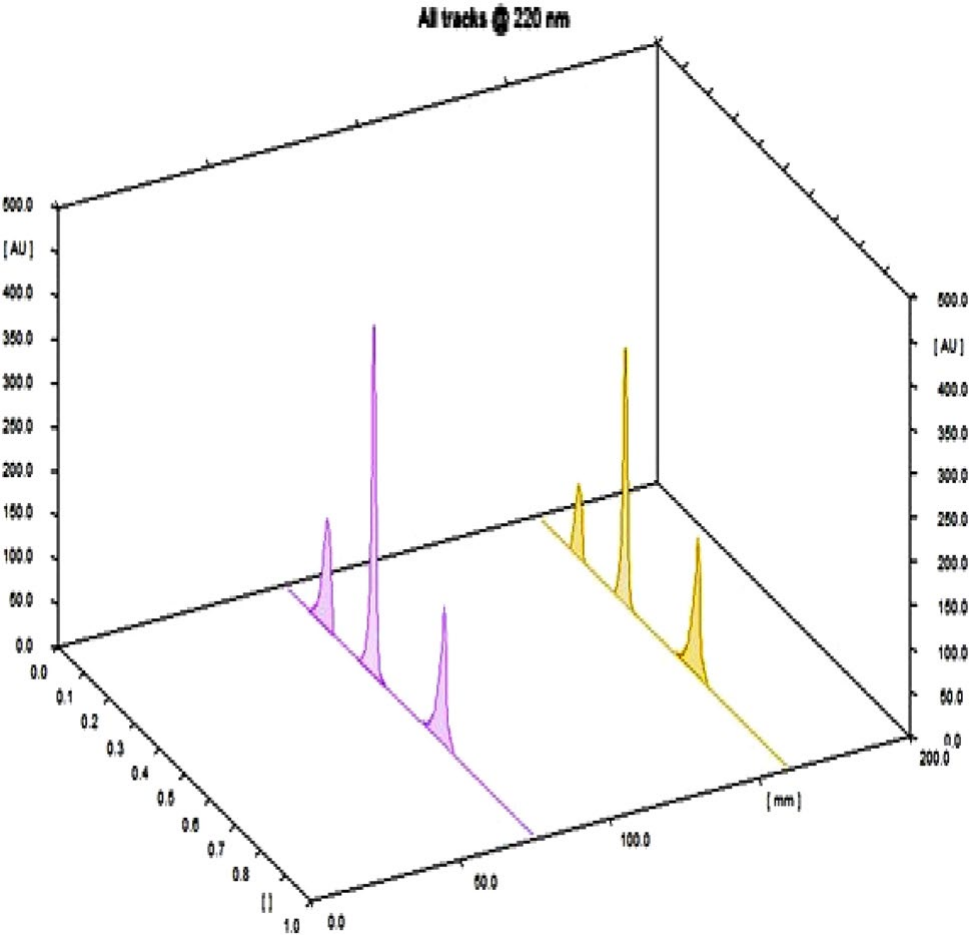
3D HP-TLC densitogram of the separated peaks from the mixture of hexythiazox (R_t_: 0.15), imidacloprid (R_t_: 0.35) and thiamethoxam (R_t_: 0.60); the elusion system is methanol:chloroform:glacial acetic acid:triethyl amine (8.5:1.5:0.2:0.1, v:v:v) and the detection wavelength is 220 nm.

Numerous detection wavelengths were selected; detection at 220 nm gave maximum sensitivity. To minimize solvent volatility and increase homogeneity, the elusion jar was saturated with the developing system for 30 min. Scored Rf values were found at 0.15, 0.35, and 0.60, correspondingly to, HTX, IDD, and TTM, as shown in Fig. 1.

### RP-HPLC methodology optimization

**Figure 2:**
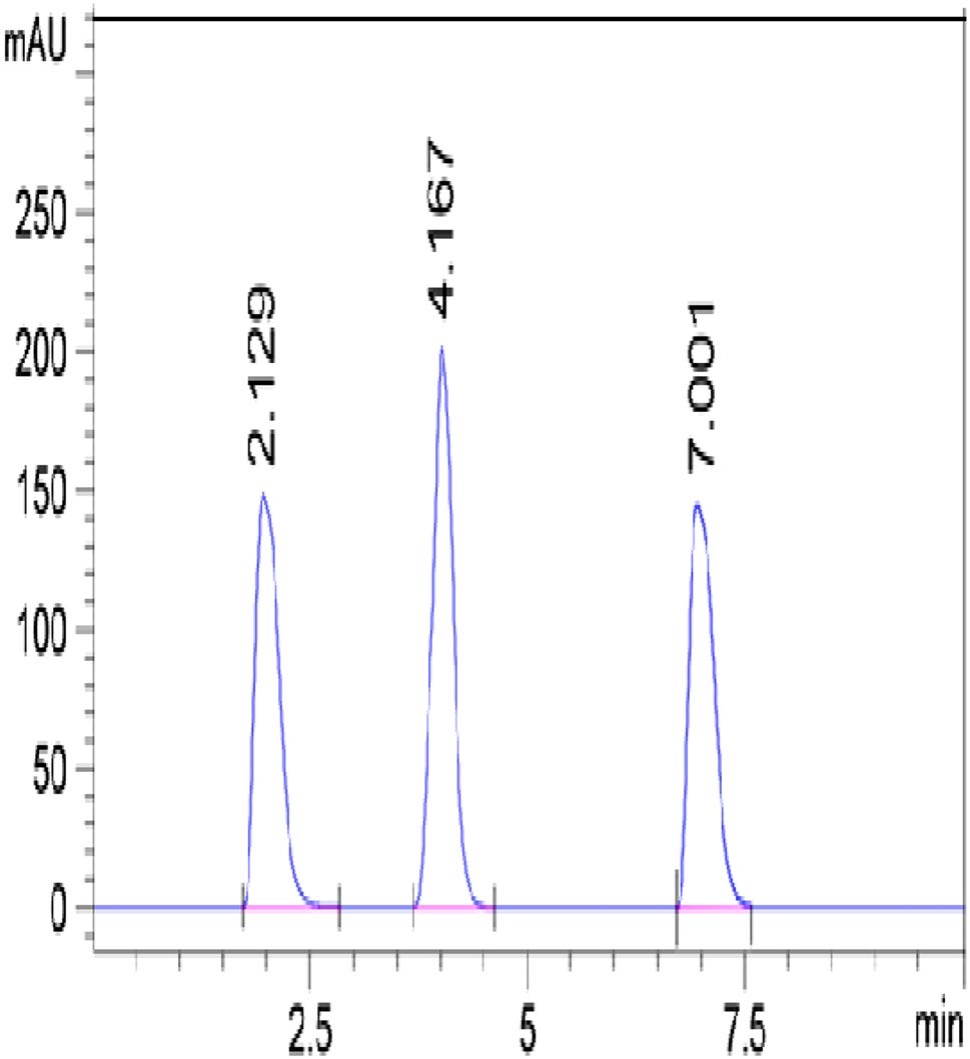
RP-HPLC chromatogram of the separated peaks from the mixture of thiamethoxam (R_t_: 2.12), imidacloprid (R_t_: 4.16) and hexythiazox (R_t_: 7.00); the elusion system is acetonitrile:water (20:80, v:v), pH adjusted to 2.8 with orthophosphoric acid and the detection wavelength is 230 nm.

Several criteria were employed to attain a superlative separation for the ternary mixture as well as detection λ wavelength and flow rate, using the C8 column. Mixtures of methanol:water in ratios 700:300 and 500:500 and methanol:0.2% acetic acid solution in ratio 800:200 were eluted but the uncompleted separation was obtained. The mixture of acetonitrile:water in a ratio of 200:800 improved the separation slightly. Adjustment of the mixture pH 2.8 with orthophosphoric acid resulted in great improvement of the peaks' resolution and well-formed peaks with no tailing. Detection of peaks at 230 nm gave a maximum sensitivity and frequently the lowest LOQ and LOD. Many flow rates were tuned; 1 mL min^−1^ produced a better resolution in a fast run. Values of 2.129, 4.167, and 7.001 min were the retention times of TTM, IDD, and HTX, respectively, as illustrated in Fig. 2. Table 1 showed the regression equation and linearity parameters for both methods.

**Table 1:** Validation assessment results of the proposed HP-TLC and RP-HPLC methods for determination of hexythiazox (HTX), imidacloprid (IDD) and thiamethoxam (TTM).

Parameter	HP-TLC method			RP-HPLC method		
	HTX	IDD	TTM	HTX	IDD	TTM
Range (µg mL^−1^ or µg band^−1^)	0.05–0.31 µg band^−1^	0.20–2.00 µg band^−1^	0.10–1.00 µg band^−1^	0.30–3.10 µg mL^−1^	2.00–20.00 µg mL^−1^	1.00–10.00 µg mL^−1^
Slope	0.9910	0.4458	0.6996	1.5040	0.3489	0.4988
Intercept	0.0132	0.0026	0.0004	0.0131	0.0199	0.0192
Correlation coefficient	0.9998	0.9998	0.9998	0.9998	0.9998	0.9999
Accuracy (mean ± SD)	99.66 ± 0.974	99.41 ± 0.950	99.89 ± 0.983	99.24 ± 0.921	99.69 ± 0.681	99.20 ± 0.692
Precision						
Repeatability^a^ (RSD %)	0.501	0.429	0.409	0.389	0.493	0.486
Intermediate precision^a^ (RSD %)	0.920	0.792	0.776	0.738	0.861	0.853
Specificity	99.18	99.24	99.72	99.48	100.09	99.68
LOD^b^	0.01	0.06	0.03	0.09	0.65	0.33
LOQ^b^	0.04	0.19	0.09	0.28	1.96	0.98
Robustness (%RSD)						
Chloroform ratio ± 1%	0.538	0.517	0.451	–	–	–
Methanol ratio ± 1%	0.428	0.418	0.384	–	–	–
Acetonitrile ratio ± 1%	–	–	–	0.506	0.483	0.410
Water ratio ± 1%	–	–	–	0.318	0.296	0.309
Flow rate ± 0.2 mL min^−1^	–	–	–	0.359	0.402	0.418
Scanning wavelength ± 1 nm	0.439	0.447	0.384	0.539	0.608	0.519
Different analyst	0.508	0.416	0.583	0.308	0.628	0.537

^a^Repeatability (n = 3), average of three different concentrations. The intermediate precision (n = 3), average of the same three different concentrations repeated three times in three successive days.

^b^Limit of detection and limit of quantitation are determined via calculations (LOD = 3.3 × SD of the response/slope, LOQ = 10 × SD of the response/slope, respectively).

### Methodology validity

The validity of both methods is verified by the following ICH rules^20^.

#### Linearity, accuracy, precision, LOD, LOQ, and specificity

After plotting the calibration curves and calculating regression equations for the concerning compounds, each method's linearity for determining the compound mixture was approved. The recovery % of blind authentic insecticide samples had been assessed and ranges of the concentration had been determined to prove accuracy. The means percentages and standard deviations of the accuracy of the HP-TLC method for the determined compounds were 99.66 ± 0.974, 99.41 ± 0.950, and 99.89 ± 0.983, correspondingly. The values were 99.24 ± 0.921, 99.69 ± 0.681, and 99.20 ± 0.692, respectively, when they were determined by the RP-HPLC method. The precision of the methods was proved by calculating the repeatability and intermediate precision values. The relative standard deviation percentages of the methods’ repeatability and intermediate precision ranged from 0.389 to 0.920. Values of the limit of detection (LOD) and limit of quantitation (LOQ) were calculated for both methods by dividing the standard deviation of the residual concentration over the slope of the calibration line of every compound in the detection ranges. LOD values of the HP-TLC and RP-HPLC methods for the concerned drugs were (0.33, 0.65, 0.09) and (0.03, 0.06, 0.01), respectively. LOQ values of them for the drugs were (0.98, 1.96, 0.28) and (0.09, 0.19, 0.04), correspondingly.

All validity parameters were illustrated in Table 1. Methods specificity was proved via the determination of R% for each compound as illustrated in the table.

#### Methods robustness

Minor changes in the mobile system content and/or saturation time were tested in the HP-TLC methodology. Also, changes in the volume of orthophosphoric acid and/or flow rates were tested for the RP-HPLC methodology. Robustness (%RSD) values ranged from 0.296 to 0.608 for both methods. No remarked effects on the retention times values, the symmetry, and the area of the peaks, Table 1.

#### Systems suitability

Peak asymmetry was measured and other parameters, like selectivity factor (α) and resolution (Rs), were calculated to evaluate the methods' system suitability. Perfect results were attained and shown in Table 2. Both methods had resolution factors of ≥ 1.78 and selectivity factors of ≥ 1.71.

**Table 2:** System suitability testing parameters of HP-TLC and HPLC methods for determination of (HTX), (IDD) and (TTM).

Parameter	HP-TLC method			HPLC method			Reference value (USP, 2011)
	HTX	IDD	TTM	HTX	IDD	TTM	
Tailing factor^a^ (T)	1.12	1.33	1.25	1.16	1.10	1.20	> 1.5
Retention factor (k): HP-TLC Capacity factor (K^'^): HPLC	5.67	1.86	0.67	4.13	2.41	1.10	1–10 for HPLC 0–10 for HP-TLC^21^
Resolution (R_S_)	2.24		2.04	2.35		1.78	< 1.5
Selectivity (α)	3.66		1.80	1.71		2.18	< 1
Column efficiency (N)	–	–	–	1855	1102	196	Increase with efficiency of the separation
HETP (cm plate^−1^)	–	–	–	0.014	0.023	0.137	The smaller the value the higher the column efficiency

*HETP* height equivalent to theoretical plate, (cm plate^−1^).

^a^Calculated using three peaks.

#### Methods applicability to commercial formulations

Acceptable recovery percentages were attained upon the application of the methods to the trade sample, Maccomite^®^ Powder, Imidamex^®^, and Pelexam^®^. The method of HP-TLC gives R%; of 99.66, 99.24 and 99.41, respectively, whereas the RP-HPLC method gives R%; of 99.69, 99.89 and 99.20, respectively (Table 3).

**Table 3:** Determination of (HTX), (IDD) and (TTM) in their commercial formulations Maccomite®, Imidamex® and Pelexam®, respectively, by the proposed HP-TLC and HPLC methods and application of the standard addition technique.

HP-TLC method					HPLC method				
Taken (µg band^−1^)	Found^a^ %	Pure added (µg band^-1^)	Pure found (µg band^−1^)	Recovery^b^ %	Taken (µg mL^−1^)	Found^a^ %	Pure added (µg mL^−1^)	Pure found (µg mL^−1^)	Recovery^b^ %
HTX									
0.10	100.09	0.05	0.05	99.76	1.00	100.13	0.50	0.50	100.98
		0.10	0.10	98.95			1.00	1.00	99.59
		0.15	0.15	100.56			2.00	1.98	99.00
Mean ± SD				99.76 ± 0.814	Mean ± SD				99.86 ± 1.021
IDD									
0.80	99.52	0.50	0.50	99.57	8.00	99.61	5.00	4.94	98.85
		0.80	0.80	100.20			8.00	8.02	100.28
		1.00	0.99	98.75			10.00	9.84	98.40
Mean ± SD				99.51 ± 0.721	Mean ± SD				99.18 ± 0.980
TTM									
0.40	99.91	0.20	0.20	100.77	3.00	99.59	2.00	1.99	99.34
		0.40	0.40	100.54			3.00	3.02	100.51
		0.60	0.60	99.25			5.00	4.94	98.86
Mean ± SD				100.19 ± 0.824	Mean ± SD				99.57 ± 0.853

^a^Average of six determinations.

^b^Average of three determinations.

### Matrix effect and application to field sample

Analysis of the field samples showed adequate values of the insecticide residues that follow the acclaimed quantity for human health and plant yield (20:22), results were listed in Table S1. The proposed technique of the field sample preparation offered in this research was related to using just the superficial part of the sprayed tomatoes. So, only the peel pieces were introduced in field sampling. A claim of the standard addition to examining the matrix effect was done and the results were illustrated in Table 4.

**Table 4:** Application of the standard addition technique to examine the matrix effect.

HP-TLC method					RP-HPLC method				
Taken (µg/band)	Found^a^%	Pure added (µg/band)	Pure found (µg/band)	Recovery %	Taken (µg/mL)	Found^a^%	Pure added (µg/mL)	Pure found (µg/band)	Recovery %
HTX									
0.10	100.09	0.05	0.05	99.76	1.00	100.13	0.50	0.50	100.98
		0.10	0.10	98.95			1.00	1.00	99.59
		0.15	0.15	100.56			2.00	1.98	99.00
Mean ± SD				99.76 ± 0.81	Mean ± SD				99.85 ± 1.02
IDD									
0.80	99.52	0.50	0.50	99.57 100.20 98.75	8.00	99.61	5.00	4.94	98.85
		0.80	0.80				8.00	8.02	98.40
		1.00	0.99				10.00	9.84	100.28
Mean ± SD				99.51 ± 0.72	Mean ± SD				99.18 ± 0.98
TTM									
0.40	99.91	0.20	0.20	100.77	3.00	99.59	2.00	1.99	99.34
		0.40	0.40	100.54			3.00	3.02	100.51
		0.60	0.60	99.25			5.00	4.94	98.86
Mean ± SD				100.19 ± 0.82	Mean ± SD				99.57 ± 0.85

^a^Average of three determinations.

### Statistical analysis

A comparative statistical analysis of the outcomes attained by the proposed methods for the analysis of the pure samples of HTX, IDD, and TTM and those attained by the published methods^17^ was carried out. It showed that there were insignificant differences between them, Table 5. Student’s t-test ranged from 0.02 to 1.51 and the values of the F-test varied from 1.18 to 2.09. But the suggested methods had significant specificity; they determined only the insecticides which were applied to the tomato fruits, definitely. Whereas, the published GC method determined 186 insecticides.

**Table 5:** Statistical comparison of the results obtained by the proposed methods and the reported method^17^.

Parameter	HP-TLC method			RP-HPLC method			Reported method		
	HTX	IDD	TTM	HTX	IDD	TTM	HTX	IDD	TTM
Mean	100.80	99.48	99.13	99.50	99.59	99.20	99.70	99.48	99.88
SD	0.99	0.97	0.87	1.05	0.83	0.93	1.14	1.11	1.26
Variance	0.98	0.94	0.76	1.10	0.67	0.86	1.30	1.23	1.59
N	7	7	7	7	7	7	7	7	7
Student’s *t*-test* (2.45)	1.06	0.02	1.51	0.90	0.22	0.62	–	–	–
*F*- test* (4.28)	1.33	1.31	2.09	1.18	1.84	1.85	–	–	–

*Figures in parenthesis are the corresponding tabulated values at p = 0.05.

### Greenness and economical efficacy of the samples preparation

The unique technique through which the analyst followed in the crop sample preparation affected greatly the volume used in the sprayed insecticides. The analyst peeled only the sprayed parts of the tomato fruits and then used them in extraction procedures instead of using the whole fruits. That had a great factor in diminishing the hazardous effect of the organic solvent waste on the environment and spending money on purchasing excessive amounts of extraction materials.

## Conclusion

The proposed methodologies, HP-TLC and RP-HPLC, can determine the insecticides' residues in the pure forms and the commercial formulations, accurately. Also, they can be applied to natural specimens. The suggested HP-TLC method has the advantage of low-cost and simple procedures. Whereas, with RP-HPLC one has the privilege of high sensitivity and speediness. Generally, the whole procedures starting from the preparation of the samples to the final process of the determination obey the current international greenness trend.

Conclusively, the developed chromatographic methods can be used for monitoring residual insecticides on tomatoes without the interference of the matrix effect and analyzing commercial formulations without the interference of excipients by green and economic procedures. Statement of Guideline Tomato fruits were cultivated and collected regarding local guidelines of the Egyptian Ministry of Agriculture and Land Reclamation^21^.

## Supplementary Information

Supplementary Information 1.

Supplementary Information 2.

## Data Availability

All data generated or analyzed during this study are included in this published article (and its supplementary information files).
