# Supplementary material for: Applying novel economic simple green sample preparation procedures on natural and industrial specimens for chromatographic determination of insecticidal residues
Source: Sci Rep. 2023 May 3;13:7209. doi: 10.1038/s41598-023-33421-7 (PMC10156696; doi:10.1038/s41598-023-33421-7)
Supplement: Supplementary file 1 — Supplementary Information 1. [file 41598_2023_33421_MOESM1_ESM.docx]

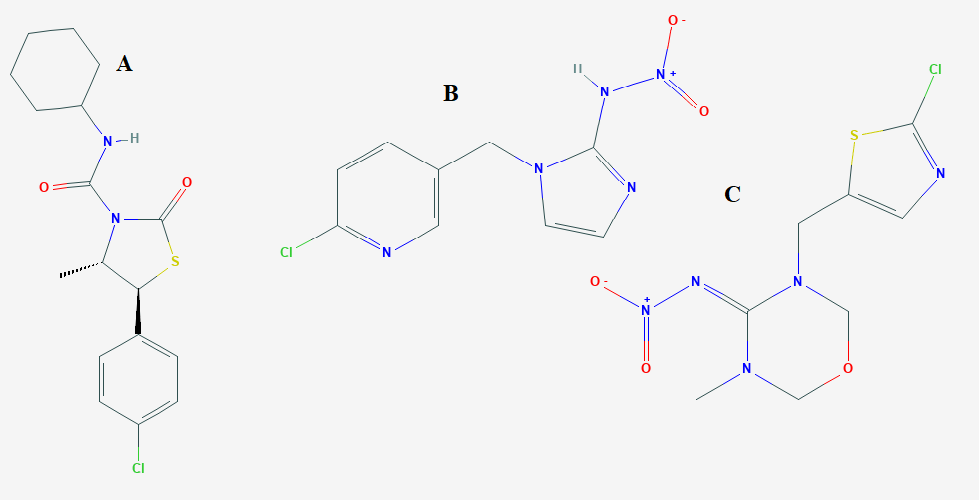


**Figure S1:** Molecular configurations of hexythiazox (A), imidacloprid (B) and thiamethoxam (C).

**Table S1:**

Application of the proposed methods to the field samples to determine residuals of hexythiazox (HTX), imidacloprid (IDD) and thiamethoxam (TTM).

| **Pesticides** | **Residue founded* (mg Kg^-1^)** | | | | **Safe dose****  **(mg Kg^-1^)** |
| --- | --- | --- | --- | --- | --- |
|  | **By day 1** | | **By day 10** | |  |
|  | **HP-TLC method** | **RP-HPLC method** | **HP-TLC method** | **RP-HPLC method** |  |
| HTX | 2.50 | 2.51 | 1.02 | 1.01 | 2.50 |
| IDD | 41.01 | 41.00 | 23.22 | 23.11 | 42.00 |
| TTM | 31.00 | 30.98 | 14.80 | 14.75 | 34.00 |

*Average of three determinations.

**Recommended dose for human and vegetable productivity [20:22].
