# Supplementary material for: Applying novel economic simple green sample preparation procedures on natural and industrial specimens for chromatographic determination of insecticidal residues
Source: Sci Rep. 2023 May 3;13:7209. doi: 10.1038/s41598-023-33421-7 (PMC10156696; doi:10.1038/s41598-023-33421-7)
Supplement: Supplementary file 2 — Supplementary Information 2. [file 41598_2023_33421_MOESM2_ESM.docx]

**Supplementary information**

In HP-TLC, samples are spotted on plates (20 × 10 cm previously washed with methanol and dried at 60°C for 5 min) using 10 µL of each prepared solution in the form of bands with a band length of 6 mm using a Camag-Linomat IV applicator, 10 mm from the bottom edge of the plate and 5 mm apart from each other. Saturated chromatographic jar with the mobile phase for 30 min is used for ascending development, scanned at 220 nm. Linearity is obtained by plotting the concentrations versus the integrated peaks area/104 in the range of 0.05– 0.31, 0.2- 2 and 0.1- 1 µg per band for HTX, IDD, and TTM, respectively, and the regression equations are calculated.

In field sample preparation, the samples of each replicate are taken after 1, 3, 7 and 10 days of the blend spraying date. Entirely, Procedures of samples preparation are done thoroughly via washed gears to avoid unintentionally transference from one substance or object to another, technically. Samples are kept cold to decrease insecticides’ loss probability following the recommendation of the European Commission.

Repeatability is assessed via analysis of the working standard solutions of HTX in three different concentrations (0.05, 0.12 and 0.22 µg/band) and of each IDD and TTM in concentrations (0.2, 0.4 and 1 µg/band) for HP-TLC method, and of HTX in concentrations (0.5, 0.9 and 2.3 µg mL-1) and of IDD and TTM in concentrations (2, 4 and 8 µg mL-1) for HPLC. They are repeated three times within the day. The intermediate precision is tested via assay of the same concerning compounds’ working solutions. They are repeated three times in three successive days.
